# Breast‐conserving therapy is associated with better survival than mastectomy in Early‐stage breast cancer: A propensity score analysis

**DOI:** 10.1002/cam4.4510

**Published:** 2022-02-25

**Authors:** Jiali Ji, Shushu Yuan, Jiawei He, Hong Liu, Lei Yang, Xuexin He

**Affiliations:** ^1^ Department of Oncology Nantong Tumor Hospital Affiliated Tumor Hospital of Nantong University Nantong Jiangsu China; ^2^ Department of breast surgery Hangzhou Hospital of Traditional Chinese Medicine Hangzhou Zhejiang China; ^3^ Department of Oncology National Cancer Center/National Clinical Research Center for Cancer/Cancer Hospital & Shenzhen Hospital, Chinese Academy of Medical Sciences and Peking Union Medical College Shenzhen China; ^4^ Department of Breast Medical Oncology The Cancer Hospital of the University of Chinese Academy of Sciences Hangzhou China; ^5^ Division of Internal Medicine The University of Texas MD Anderson Cancer Center Houston Texas USA

**Keywords:** breast‐conserving therapy, mastectomy, survival, tumor subtypes

## Abstract

**Background:**

Recent retrospective studies have reported that breast‐conserving therapy (BCT) led to improved overall survival (OS) than mastectomy in some populations. We aimed to compare the efficacy of BCT and mastectomy using the SEER database.

**Materials and methods:**

Between 2010 and 2015, 99,790 eligible patients were identified. We included early‐stage breast cancer patients with 5cm or smaller tumors and three or fewer positive lymph nodes in our study. We compared the OS and breast cancer‐specific survival (BCSS) results among patients with BCT and those with mastectomy. Kaplan‐Meier plots, Cox proportional hazard regressions, competing risk analysis, and multivariate regressions were used to evaluate the outcomes. Propensity‐score matching was used to assemble a cohort of patients with similar baseline characteristics.

**Results:**

In our study, 77,452 (77.6%) patients underwent BCT and 22,338 (22.4%) underwent mastectomy. The 5‐year OS rate was 94.7% in the BCT group and 87.6% in the mastectomy group, and the 5‐year BCSS was 97.2% in the BCT and 94.3% in the mastectomy group. Multivariate analysis in the matched cohort showed that women underwent mastectomy was associated with worse OS (Hazard ratio (HR) = 1.79; 95% confidence intervals (CIs) = 1.59–2.02, *p* < 0.001) and BCSS (HR = 1.88; 95% CIs = 1.61–2.18, *p* < 0.001) results compared with those underwent BCT. Patients with different subtypes and age group (>50 years old; ≤50 years old) received BCT showed significantly better OS and BCSS results than those received mastectomy. The effect of surgery choice on survival yielded similar results either for all patients or matched cohorts.

**Conclusions:**

Our study showed that BCT was associated with improved survival compared with mastectomy in early‐stage breast cancer patients. It seems advisable to encourage patients to receive BCT rather than mastectomy in early‐stage patients when feasible and appropriate.

## Graphical abstract

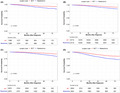

## 1 INTRODUCTION

Breast cancer is the most common neoplasm found among women worldwide.^1^ Breast‐conserving therapy (BCT) and mastectomy are the most common locoregional treatments for early or locally advanced breast cancer.^2^ BCT refers to breast‐conserving surgery followed by radiotherapy to eradicate any microscopic residual disease. In addition to being cosmetically acceptable, it also offers equivalent survival rates.^3^

The long‐term effects of BCT have been evaluated in multiple clinical trials comparing the overall survival (OS), local, and regional recurrence with mastectomy over the past decades. In the NSABP B‐06 trial, a lower ipsilateral breast cancer recurrence rate was observed in patients with tumors less than 4 cm in size following BCT than those who had a mastectomy. However, there were no significant differences in OS rates between the groups.^4^ Using the Surveillance, Epidemiology and End Results (SEER) database from 1998 to 2008, a recent analysis compared the OS result of patients who underwent mastectomy, mastectomy with radiation, and BCT. According to their findings, patients who underwent BCT had higher survival rates than those who underwent mastectomy or mastectomy with radiation matching for tumor size and lymph node location.^5^ Based on a registry‐based follow‐up study involving 6,387 breast cancer patients, there is a benefit of BCT over mastectomy for patients with stage T1N1M0. In other stages of breast cancer, there were no survival benefit.^6^ Previous studies, however, did not have access to tumor subtypes, and had few samples, making them susceptible to selection bias. Traditionally, BCT had been underutilized due to surgeon and patient preference.^7^ There is an increasing need to re‐examine survival outcomes for mastectomy and BCT, in order to inform an optimal surgery choice for an individual patient, especially with the development of radiotherapy techniques that can eliminate micrometastases.^8^ We compared the OS and BCSS rates between BCT and mastectomy in a large number of early‐stage breast cancer patients in our study. We further explored survival outcomes in breast cancer patients stratified by tumor subtypes, age, tumor, and lymph node stage in the SEER database.

## 2 MATERIALS AND METHODS

### 2.1 Study design

It is a retrospective cohort study consisting of breast cancer patients from the SEER cancer registries between 2010 and 2015. The data on patients’ demographics, vital status, tumor characteristics, treatment, and survival times were gathered using SEERStat software. The follow‐up cutoff was on December 31, 2019. In order to compare the survival results of standard BCT and mastectomy, we focused on patients with invasive ductal carcinoma who received either lumpectomy with radiotherapy or mastectomy with or without radiotherapy.

### 2.2 Participants

We identified eligible cases based on the following criteria: female, age between 18–80 years old, unilateral breast cancer, pathologically diagnosed, with primary breast cancer, with a tumor size of 5 cm or smaller, with three or fewer positive lymph nodes, received surgery (lumpectomy (site‐specific surgery codes 20–24) with radiation, mastectomy (site‐specific surgery codes 41,50–51, 80)), and without metastasis at diagnosis. The stage was based on the 7th edition of the AJCC Cancer Staging Manual.

**FIGURE 1:**
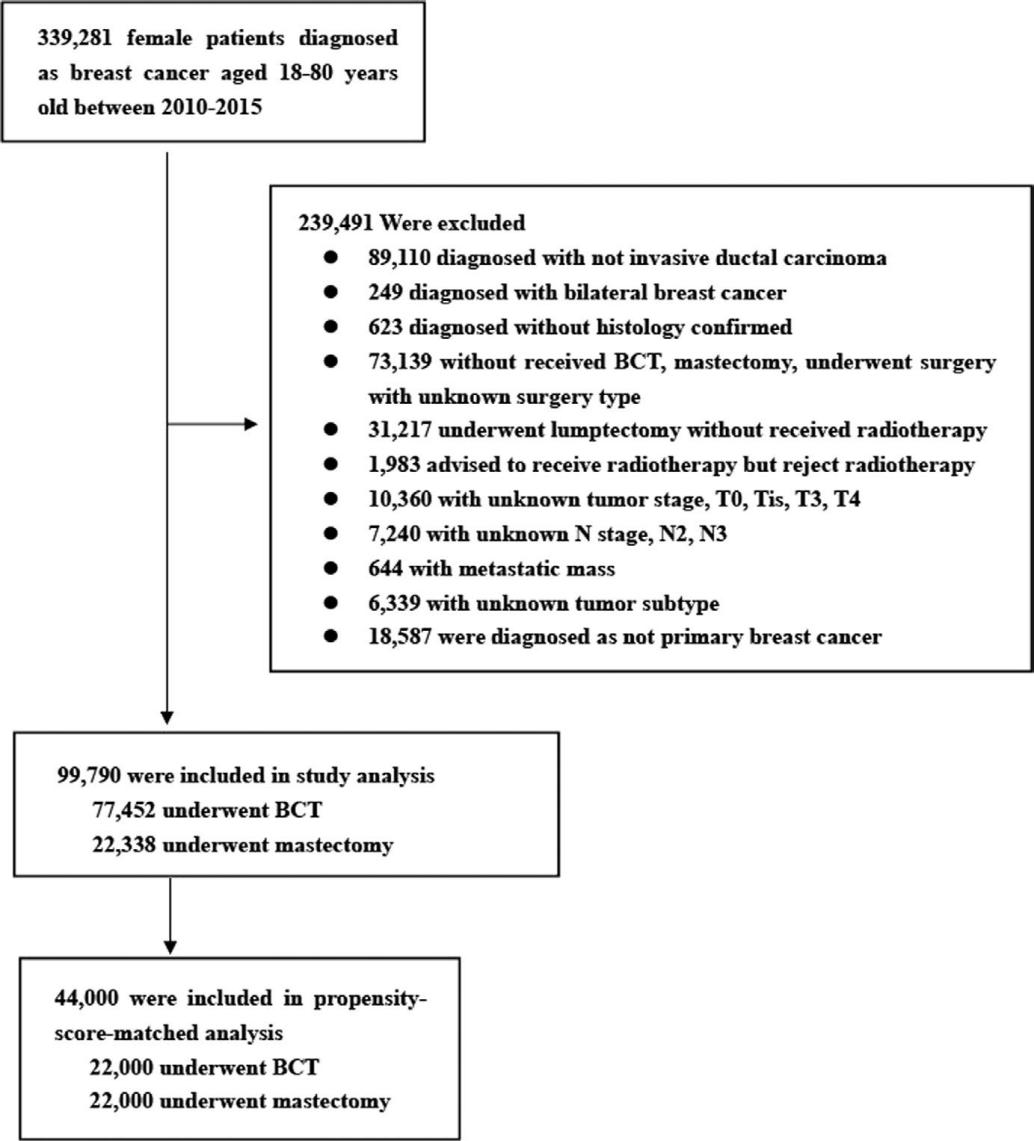
Flow chart of patient selection

The exclusion criteria were (1) not invasive ductal carcinoma (*n* = 89,110); (2) bilateral tumor (*n* = 249); (3) without histologically confirmed (*n* = 623); (4) without underwent lumpectomy, mastectomy, underwent surgery with unknown surgery type (*n* = 73,139); (5) underwent lumpectomy without received radiotherapy (*n* = 31,217); (6) advised to receive radiotherapy but reject (*n* = 1,983); (7) tumor stage T0, Tis, T3,T4, or unknown (*n* = 10,360); (8) lymph node stage N2, N3, or unknown (*n* = 7,240); (9) with distant metastasis (*n* = 644); (10) with unknown tumor subtypes (*n* = 6,339); (11) not primary breast cancer (*n* = 18,587). In our study, patients with >3 positive lymph nodes were excluded because these patients would be more likely to be indicated to receive radiation therapy regardless of surgery type, and since our study focused on patients with early breast cancer. Figure 1 shows the flowchart of inclusion. In our study, the primary endpoint was overall survival (OS). OS was calculated from the date of diagnosis as breast cancer to death for any cause or last follow‐up time. The secondary outcome was breast cancer‐specific survival (BCSS) from the date of diagnosis to the date of death caused by breast cancer.

### 2.3 Statistical analysis

Pearson's chi‐square test was used to compare the clinical pathology between the BCT and mastectomy groups. To balance the different characteristics between each group, propensity‐score matching (PSM) was used with a ratio of 1.0. The PSM method matches a treatment case with one or more control cases based on their propensity scores, reducing the selection bias in the observational studies of causal arguments.^9^ In each case, a caliper width equal to 0.001 of the standard deviation of the logit of the propensity score was used. Matching was performed using the MatchIt package. In our study, matching was based upon age, tumor stage, node stage, race distribution, nuclear grade, and tumor subtype. The Kaplan–Meier method was used to estimate the OS rate and plot survival curves. Our study used the log‐rank test to identify factors associated with OS across different patient groups. Cox proportional‐hazards regression models evaluated the hazard ratios (HRs) with 95% confidence intervals (CIs) for OS results. Fine‐Gray competing risk analysis and multivariate regression model of competing risk were used to evaluate prognostic factors for BCSS.

Using Cox regression model and competing risk analysis stratified by tumor and lymph node stage in all cohort, we further evaluated the comparative risks and benefits of OS and BCSS results, respectively. In addition, Kaplan–Meier method, multivariate Cox models, Fine‐Gray competing risk analysis, and multivariate regression model of competing risk were also done on the propensity‐matched sample. All statistical analyses and survival plots were done using SPSS 22.0 (IBM Corporation) and R software (Version 3.6.1, R Foundation for Statistical Computing. http://www.R‐project.org/). In our study, a *P*‐value <0.05 was considered as statistically significant.

## 3 RESULTS

### 3.1 Demographic and matched characteristics

A total of 99,790 patients with primary breast cancer who met the criteria were eventually selected. A total of 77,452 patients (76.6%) underwent BCT, while 22,338 (22.4%) underwent mastectomy. Patients’ demographics and tumor characteristics are shown in Table 1. BCT and mastectomy groups had significantly different distributions of all variables (*p* < 0.001). In propensity‐score matching, 22,000 patients who underwent mastectomy were matched with 22,000 patients who underwent BCT.

**TABLE 1:** Patient‐, tumor‐, and treatment‐related characteristics in our cohort

	Before Matching				*P*	After Matching				*P*
	BCT		Mastectomy			BCT		Mastectomy		
	*n*	%	*n*	%		*n*	%	*n*	%	
Age (years)					0.001					0.104
≤50	16190	20.9	4905	22.0		4997	22.7	4854	22.1	
>50	61262	79.1	17433	78.0		17003	77.3	17146	77.9	
T stage					<0.001					0.954
T1	60337	77.9	12409	55.6		12364	56.2	12357	56.2	
T2	17115	22.1	9929	44.4		9636	43.8	9643	43.8	
N stage					<0.001					0.952
N0	63707	82.3	14578	65.3		14506	65.9	14513	66.0	
N1	13745	17.7	760	34.7		7494	34.1	7487	34.0	
AJCC 7th stage					<0.001					0.094
Ⅰ	54826	70.8	10151	45.4		10309	46.9	10133	46.1	
Ⅱ	22626	29.2	12187	54.6		11691	53.1	11867	53.9	
Race					<0.001					0.071
White	62031	80.1	15824	70.8		15783	71.7	15791	71.8	
Black	7939	10.3	2735	12.2		2857	13.0	2711	12.3	
Other	7085	9.1	3665	16.4		3252	14.8	3395	15.4	
Unknown	397	0.5	114	0.5		108	0.5	103	0.5	
Tumor subtype					<0.001					1.000
HR−/HER2+ (HER2‐enriched)	2481	3.2	1507	6.7		1396	6.3	1396	6.3	
HR+/HER2− (Luminal A)	59956	77.4	14817	66.3		14710	66.9	14710	66.9	
HR+/HER2+ (Luminal B)	7063	9.1	3036	13.6		2970	13.5	2970	13.5	
HR−/HER2− (Triple‐negative)	7952	10.3	2978	13.3		2924	13.3	2924	13.3	
Chemotherapy					<0.001					<0.001
No/unknown	49988	64.5	11856	53.1		10653	48.4	11780	53.5	
Yes	27464	35.5	10482	46.9		11347	51.6	10220	46.5	
Nuclear grade					<0.001					0.998
I/II	54176	69.9	13075	58.5		12959	58.9	12952	58.9	
III/Ⅳ	21702	28.0	8750	39.2		8570	39.0	8577	39.0	
Unknown	1574	2.0	513	2.3		471	2.1	471	2.1	
Radiation therapy					‐					‐
No/unknown	0	0.0	18157	81.2		0	0.0	17947	81.5	
Yes	77452	100.0	4181	18.7		22000	100.0	4053	18.4	
Total	77452	100.0	22338	100.0		22000	100.0	22000	100.0	

Abbreviation: BCT, breast‐conserving therapy.

In the matched cohort, there was no statistically significant difference in the distribution of baseline variables besides radiotherapy and chemotherapy. In our study, patients who underwent lumpectomy must receive radiotherapy. The distribution of related variables is shown in Table 1.

### 3.2 Survival analysis before matching

**FIGURE 2:**
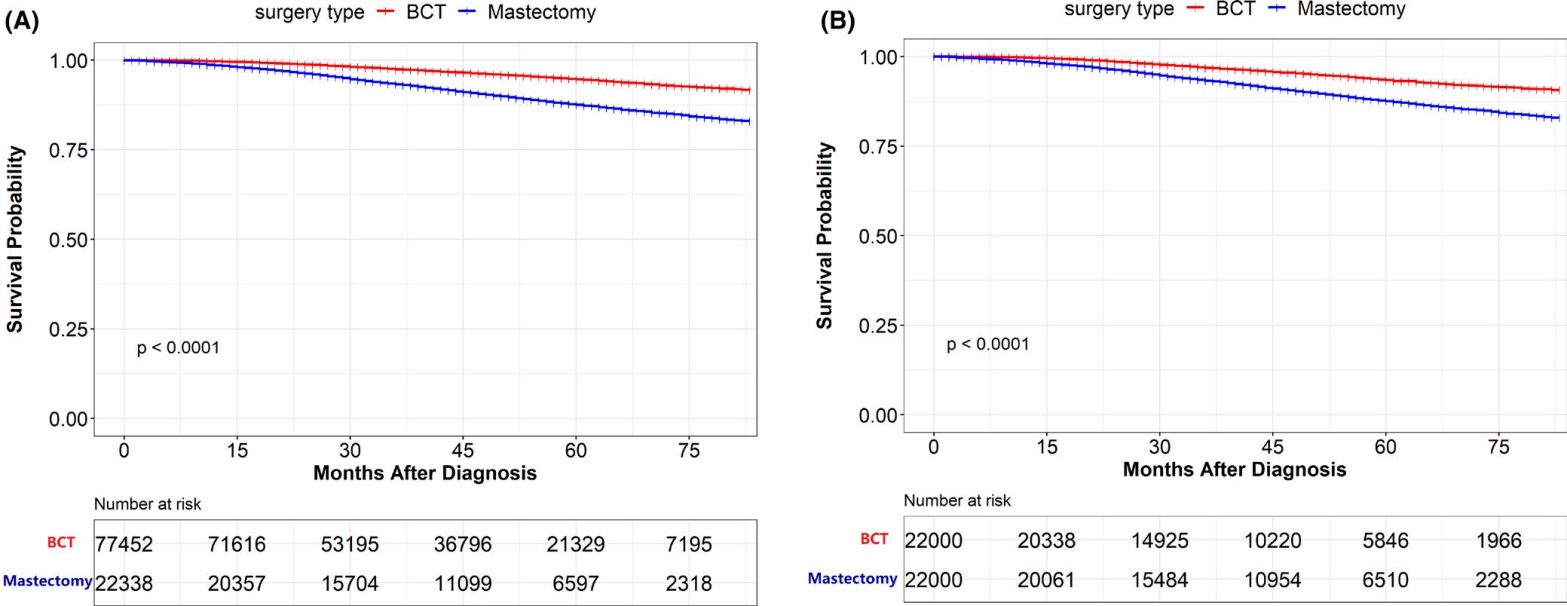
Overall survival (OS) rate of patients underwent BCT and mastectomy in all eligible patients and propensity‐score matching (PSM) cohort. (A) OS rate of patients underwent BCT and mastectomy in all eligible patients. (B) OS rate of patients underwent BCT and mastectomy in PSM cohort. BCT, breast‐conserving therapy

There were 2,850 (3.7%) deaths observed in the BCT group in all eligible breast cancer patients and 2,080 (9.3%) among patients underwent a mastectomy. BCT was proved a superior survival result compared with the mastectomy group (94.7% compared with 87.6%, *p* < 0.001). The survival plots are shown in Figure 2A. According to the Kaplan–Meier analysis, the surgery type choice, age, tumor stage, lymph node stage, race, tumor subtypes, chemotherapy, and radiotherapy are important prognostic factors for OS in breast cancer patients. Adjusting the significant factors in Kaplan–Meier analysis, Cox proportional hazards multivariate analysis showed that mastectomy (Hazard ratio (HR) = 1.78; 95% confidence intervals (CIs) = 1.59–1.98; *p* < 0.001), age over 50 years old (HR = 1.90; 95% CI = 1.75–2.07; *p* < 0.001), T2 stage (HR = 1.82; 95% CI = 1.71–1.94; *p* < 0.001), N1 stage (HR = 1.59; 95% CI = 1.49–1.70; *p* < 0.001), Black race (HR = 1.23; 95% CI = 1.14–1.33; *p* < 0.001), triple‐negative subtype (HR = 1.63; 95% CI = 1.43–1.86; *p* < 0.001), without chemotherapy (HR = 1.59; 95% CI = 1.49–1.71; *p* < 0.001), and nuclear grade Ⅲ/Ⅳ (HR = 1.62; 95% CI = 1.51–1.73; *p* < 0.001) were associated with higher risk of death (Table 2).

**TABLE 2:** Univariate and multivariate analysis of overall survival (OS) and breast cancer‐specific survival (BCSS) in all patients

Covariate	OS				BCSS			
	Univariate analysis		Multivariate analysis		Univariate analysis		Multivariate analysis	
	5‐year OS (95% CI) (%)	*P*	HR (95% CI)	*P*	5‐year BCSS (95% CI) (%)	*P*	HR (95% CI)	*P*
Surgery type		<0.001				<0.001		
BCT	94.7 (94.4–94.9)		1.0 [reference]		97.2 (97.1–97.4)		1.0 [reference]	
Mastectomy	87.6 (87.0–88.1)		1.78 (1.59–1.98)	<0.001	94.3 (93.9–94.7)		1.81 (1.58–2.08)	<0.001
Age (years)		<0.001				<0.001		
≤50	95.5 (95.1–95.8)		1.0 [reference]		99.1 (98.9–99.3)		1.0 [reference]	
>50	92.3 (92.1–92.6)		1.90 (1.75–2.07)	<0.001	95.9 (95.7–96.0)		1.24 (1.12–1.37)	<0.001
T stage		<0.001				<0.001		
T1	94.8 (94.6–95.1)		1.0 [reference]		96.9 (96.7–97.0)		1.0 [reference]	
T2	88.0 (87.5–88.5)		1.82 (1.71–1.94)	<0.001	95.7 (95.4–96.0)		2.34 (2.13–2.57)	<0.001
N stage		<0.001						
N0	94.0 (93.8–94.3)		1.0 [reference]		96.6 (96.4–96.7)	0.580	1.0 [reference]	
N1	89.2 (88.7–89.8)		1.59 (1.49–1.70)	<0.001	96.5 (96.2–96.9)		1.99 (1.81–2.19)	<0.001
Race		<0.001				<0.001		
White	93.1 (92.9–93.3)		1.0 [reference]		96.4 (96.2–96.6)		1.0 [reference]	
Black	89.6 (88.8–90.4)		1.23 (1.14–1.33)	<0.001	95.9 (95.4–96.4)		1.28 (1.15–1.42)	<0.001
Other	95.4 (94.9–96.0)		0.59 (0.53–0.66)	<0.001	98.0 (97.7–98.4)		0.61 (0.52–0.71)	<0.001
Unknown	99.7 (99.0–100.0)		0.09 (0.02–0.35)	<0.001	‐		0.09 (0.01–0.68)	<0.001
Tumor subtype		<0.001				0.140		
HR−/HER2+ (HER2‐enriched)	90.0 (88.9–91.4)		1.0 [reference]		96.6 (95.8–97.3)		1.0 [reference]	
HR+/HER2− (Luminal A)	94.2 (94.0–94.4)		0.79 (0.69–0.90)	0.206	96.6 (96.4–96.7)		0.55 (0.46–0.66)	<0.001
HR+/HER2+ (Luminal B)	94.0 (93.3–94.6)		0.73 (0.62–0.85)	<0.001	97.0 (96.5–97.4)		0.50 (0.40–0.62)	<0.001
HR−/HER2− (Triple‐negative)	85.3 (84.4–86.1)		1.63 (1.43–1.86)	<0.001	96.2 (95.7–96.6)		1.92 (1.62–2.27)	<0.001
Chemotherapy		<0.001				<0.001		
No/unknown	93.7 (93.4–93.9)		1.59 (1.49–1.71)	<0.001	95.7 (95.5–95.9)		0.79 (0.70–0.88)	<0.001
Yes	91.9 (91.6–92.3)		1.0 [reference]		97.9 (97.7–98.1)		1.0 [reference]	
Nuclear grade		<0.001				0.780		
I/II	94.9 (94.6–95.1)		1.0 [reference]		96.6 (96.4–96.8)			
III/Ⅳ	89.0 (88.6–89.5)		1.62 (1.51–1.73)	<0.001	96.5 (96.2–96.7)			
Unknown	92.2 (90.6–93.7)		1.39 (1.15–1.68)	<0.001	96.4 (95.3–97.3)			
Radiation therapy		<0.001				<0.001		0.370
No/unknown	87.8 (87.2–88.5)		1.12 (1.43–1.86)	0.052	93.9 (93.4–94.3)		1.07 (0.93–1.23)	
Yes	94.2 (94.0–94.4)		1.0 [reference]		97.2 (97.0–97.3)		1.0 [reference]	

Note

The univariate analysis was univariate Kaplan–Meier analysis in the OS and Fine‐Gray competing risk analysis in the BCSS. The multivariate analysis was Cox proportional hazards multivariate analysis in the OS and multivariate regression model of competing risk analysis in the BCSS.

Abbreviation: BCT, breast‐conserving therapy.

In multivariate analysis, other races (HR = 0.59; 95% CI 0.53–0.66; *p* < 0.001) were associated with improved OS compared with the White race. Luminal B (HR = 0.73; 95% CI = 0.62–0.85; *p* < 0.001) was proved to have a better OS result than the HER2‐positive breast cancer. While without receiving radiotherapy did not significantly impact the survival rates of breast cancer patients (HR = 1.12, CI = 1.43–1.86, *p* = 0.052).

In BCSS analysis, the 5‐year BCSS rate was 97.2% in the BCT group and 94.3% in the mastectomy group. After adjusting for relevant factors in univariate analyses, patients with mastectomy had worse prognosis (HR = 1.81, CI = 1.58–2.08, *p* < 0.001).

In the subgroup analysis of tumor subtype, age subgroup, tumor, and lymph node stage, we found that the BCT group had better OS and BCSS results than the mastectomy group (Table 3). Tumor subtypes other than HER2‐positive showed significantly better BCSS results in the BCT group compared to the mastectomy group. The survival plots of subgroup analysis are shown in Figures S1–S3.

**TABLE 3:** The overall survival (OS) and breast cancer‐specific cancer (BCSS) rate in all patients subgroup

Subgroup	BCT	Mastectomy	P	BCT	Mastectomy	P
	5‐year OS (95% CI) (%)	5‐year OS (95% CI) (%)		5‐year BCSS (95% CI) (%)	5‐year BCSS (95% CI) (%)	
T stage						
T1	95.7 (95.5–95.9)	90.9 (90.3–91.6)	<0.001	97.4 (97.2–97.5)	94.6 (94.1–95.1)	<0.001
T2	90.9 (90.3–91.5)	83.3 (82.4–84.2)	<0.001	96.8 (96.4–97.2)	93.9 (93.3–94.5)	
N stage						
N0	95.2 (95.0–95.5)	89.1 (88.5–89.8)	<0.001	97.2 (97.0–97.4)	93.9 (93.4–94.4)	<0.001
N1	92.0 (91.3–92.6)	84.7 (83.6–85.7)	<0.001	97.5 (97.1–97.8)	95.0 (94.4–95.6)	<0.001
Age						
≤50	96.4 (96.0–96.8)	92.6 (91.6–93.5)	<0.001	99.3 (99.1–99.4)	98.6 (98.1–99.0)	<0.001
>50	94.2 (93.9–94.5)	86.2 (85.5–86.8)	<0.001	96.7 (96.5–96.7)	93.1 (92.6–93.6)	<0.001
Tumor subtype						
HR−/HER2+ (HER2‐enriched)	92.2 (90.8–93.7)	87.0 (84.8–89.2)	<0.001	97.1 (96.1–97.9)	96.0 (94.6–97.1)	0.140
HR+/HER2− (Luminal A)	95.5 (95.2–95.7)	89.4 (88.7–90.0)	<0.001	97.2 (97.0–97.4)	94.0 (93.5–94.5)	<0.001
HR+/HER2+ (Luminal B)	95.8 (95.1–96.4)	90.0 (88.6–91.5)	<0.001	97.7 (97.2–98.2)	95.4 (94.3–96.3)	<0.001
HR−/HER2− (Triple‐negative)	88.6 (87.7–89.5)	76.7 (74.8–78.6)	<0.001	97.1 (96.6–97.6)	93.8 (92.6–94.8)	<0.001

### 3.3 Survival analysis after matching

After matching, the median follow‐up time was 46.0 months (interquartile range, IQR 45.6–46.4 months), and 3,005 people died from all causes. There were 953 (4.33%) death events observed in the BCT group and 2,052 (9.33%) in the mastectomy group. In the BCT group, the 5‐year OS rate was 93.4%, while in the mastectomy group it was 87.6% (*p* < 0.001) (Table 4). Based on Kaplan–Meier survival estimates, patients who underwent BCT had a better OS result than patients who underwent mastectomy. The log‐rank test p‐value was <0.001 at 5‐year points (Figure 2B). On univariate analysis, age at diagnosis, tumor stage, node stage, race, tumor subtype, nuclear grade, and radiotherapy were significantly associated with OS (Table 4). All univariate factors associated with OS were included in the multivariable Cox model. In multivariable analysis, mastectomy was associated with worse OS results than BCT (HR = 1.79; 95% CI = 1.59–2.02, *p* < 0.001). The 5‐year BCSS rate was 97.4% and 94.3% in the BCT and mastectomy group, respectively. After adjusting for the age, tumor stage, lymph node stage, race distribution, tumor subtype, chemotherapy, and radiotherapy, patients with mastectomy (vs. BCT (reference); HR = 1.88; 95% CI = 1.61–2.18, *p* < 0.001) had worse prognosis in BCSS. In conclusion, the PSM cohort demonstrated a survival benefit of BCT. The better survival result of BCT was consistent with the result in the eligible cohort.

**TABLE 4:** Univariate and multivariate analysis of overall survival (OS) and breast cancer‐specific survival (BCSS) in matched cohort

Covariate	OS				BCSS			
	Univariate analysis		Multivariate analysis		Univariate analysis		Multivariate analysis	
	5‐year OS (95% CI) (%)	*P*	HR (95% CI)	*P*	5‐year BCSS (95% CI) (%)	*P*	HR (95% CI)	*P*
Surgery type		<0.001				<0.001		
BCT	93.4 (92.9–93.8)		1.0 [reference]		97. 4 (97.1–97.6)		1.0 [reference]	
Mastectomy	87.6 (87.0–88.1)		1.79 (1.59–2.02)	<0.001	94.3 (93.9–94.7)		1.88 (1.61–2.18)	<0.001
Age (years)		<0.001				<0.001		
≤50	93.6 (93.0–94.3)		1.0 [reference]		98.9 (98.5–99.1)		1.0 [reference]	
>50	89.4 (89.0–89.8)		1.94 (1.75–2.15)	<0.001	94.9 (94.5–95.2)		1.22 (1.08–1.38)	0.001
T stage		<0.001				0.012		
T1	93.1 (92.6–93.5)		1.0 [reference]		96.0 (95.7–96.3)		1.0 [reference]	
T2	86.9 (86.2–87.5)		1.68 (1.56–1.82)	<0.001	95.4 (95.0–95.8)		2.36 (2.10–2.65)	<0.001
N stage		<0.001				0.080		
N0	91.8 (91.4–92.2)		1.0 [reference]		95.6 (95.3–95.9)		1.0 [reference]	
N1	87.6 (86.9–88.3)		1.45 (1.34–1.56)	<0.001	96.1 (95.7–96.5)		1.98 (1.77–2.21)	<0.001
Race		<0.001				<0.001		
White	90.0 (89.6–90.5)		1.0 [reference]		95.5 (95.1–95.7)		1.0 [reference]	
Black	87.0 (85.9–88.2)		1.19 (1.08–1.31)	<0.001	95.1 (94.3–95.7)		1.23 (1.08–1.39)	0.002
Other	94.7 (94.0–95.4)		0.56 (0.49–0.64)	<0.001	97.9 (97.4–98.3)		0.60 (0.50–0.72)	<0.001
Unknown	—	—	—	—	—	—		
Tumor subtype		<0.001				0.001		
HR−/HER2+ (HER2‐enriched)	90.3 (88.8–91.7)		1.0 [reference]		96.8 (95.9–97.6)		1.0 [reference]	
HR+/HER2− (Luminal A)	91.7 (91.3–92.1)		0.99 (0.85–1.16)	0.960	95.6 (95.2–95.9)		0.59 (0.48–0.72)	<0.001
HR+/HER2+ (Luminal B)	92.9 (92.0–93.8)		0.75 (0.63–0.90)	0.01	96.6 (95.9–97.2)		0.52 (0.40–0.66)	<0.001
HR−/HER2− (Triple‐negative)	81.5 (80.2–82.7)		1.83 (1.56–2.14)	<0.001	95.5 (94.8–96.2)		2.08 (1.71–2.54)	<0.001
Chemotherapy		0.600				<0.001		
No/unknown	90.5 (90.0–91.0)		‐		94.0 (93.6–94.4)		0.95 (0.84–1.08)	0.460
Yes	90.3 (89.8–90.8)		‐		97.5 (97.2–97.8)		1.0 [reference]	
Nuclear grade		<0.001				0.218		
I/II	92.8 (92.4–93.2)		1.0 [reference]		95.8 (95.4–96.0)		‐	
III/Ⅳ	86.9 (86.2–87.5)		1.42 (1.31–1.55)	<0.001	95.9 (95.4–96.2)		‐	
Unknown	89.0 (86.3–91.8)		1.35 (1.06–1.73)	0.02	95.7 (93.8–97.2)			
Radiation therapy		<0.001				<0.001		0.980
No/unknown	87.8 (87.2–88.5)		1.22 (1.09–1.37)	<0.001	93.9 (93.4–94.3)		1.00 (0.87–1.16)	
Yes	92.2 (91.8–92.6)		1.0 [reference]		97.1 (96.9–97.4)		1.0 [reference]	

Note

The univariate analysis was univariate Kaplan–Meier analysis in the OS and Fine‐Gray competing risk analysis in the BCSS. The multivariate analysis was Cox proportional hazards multivariate analysis in the OS and multivariate regression model of competing risk analysis in the BCSS. The propensity score‐matched cohort included 22,000 patients in the BCT group and 22,000 patients in the mastectomy group.

Abbreviation: BCT, breast‐conserving therapy.

**FIGURE 3:**
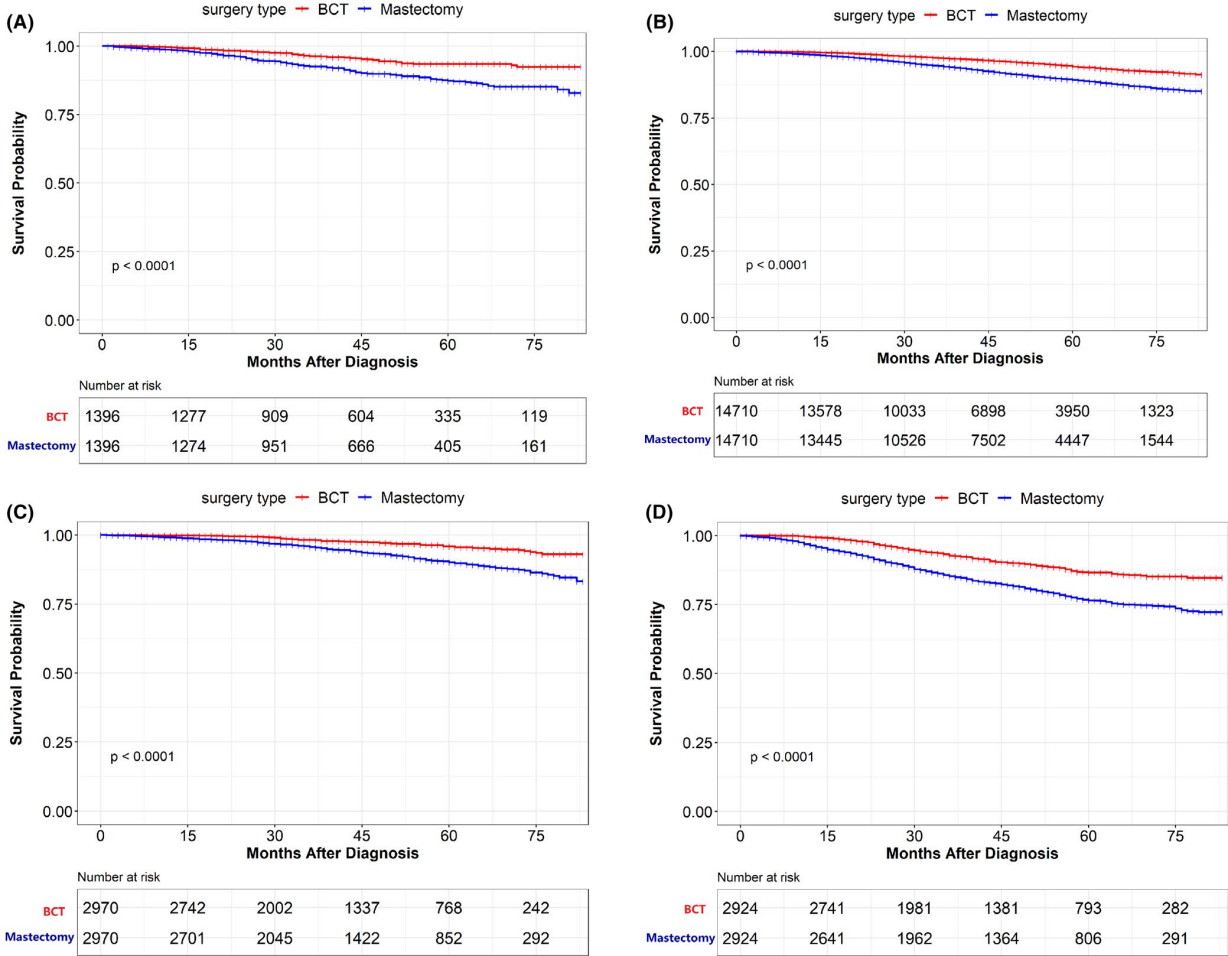
Overall survival (OS) rate of patients underwent BCT and mastectomy in the propensity‐score matching (PSM) cohort stratified in tumor subtype. (A) OS rate of patients underwent BCT and mastectomy in HER2+ breast cancer. (B) OS rate of patients underwent BCT and mastectomy in Luminal A breast cancer. (C) OS rate of patients underwent BCT and mastectomy in Luminal B breast cancer. (D) OS rate of patients underwent BCT and mastectomy in Triple‐negative breast cancer

**FIGURE 4:**
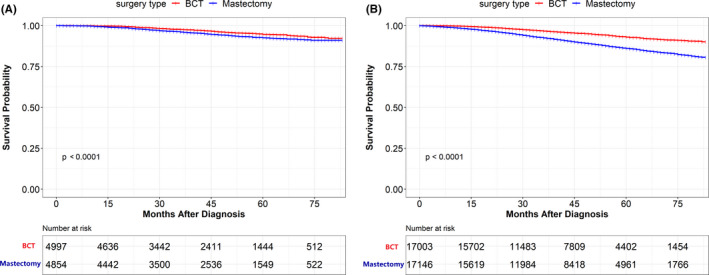
Overall survival (OS) rate of patients underwent BCT and mastectomy in the propensity‐score matching (PSM) cohort stratified in the different age groups. (A) OS rate of patients underwent BCT and mastectomy aged ≤50 years old breast cancer. (B) OS rate of patients underwent BCT and mastectomy aged >50 years old breast cancer

Subgroup analyses were conducted to determine the effect of surgery type choice on survival among patients with distinctive characteristics. Patients treated with BCT for different subtypes showed significantly improved overall survival compared to those treated with mastectomy. The Kaplan–Meier survival plots are shown in Figure 3. The BCT group showed improved OS in patients from different age subgroups as well (Figure 4). In addition, patients with tumor stages T1, T2, and node stages N0, N1 had improved survival results in the BCT group (Table 5). Figure S4 shows survival plots for different stages of patients. For BCSS analysis, patients with the BCT also had better survival results than those with mastectomy in the subgroup of different tumor subtypes, age group, tumor stages, and lymph node stages.

**TABLE 5:** The overall survival (OS) and breast cancer‐specific cancer (BCSS) rate in matched cohort patients subgroup

Subgroup	BCT	Mastectomy	P	BCT	Mastectomy	P
	5‐year OS (95% CI) (%)	5‐year OS (95% CI) (%)		5‐year BCSS (95% CI) (%)	5‐year BCSS (95% CI) (%)	
T stage						
T1	95.4 (94.8–95.9)	90.9 (90.3–91.6)	<0.001	97.5 (97.1–97.9)	94.6 (94.1–95.1)	<0.001
T2	90.8 (90.0–91.6)	83.1 (82.2–84.1)	<0.001	97.2 (96.7–97.6)	93.8 (93.2–94.)	<0.001
N stage						
N0	94.7 (94.2–95.2)	89.1 (88.5–89.8)	<0.001	97.3 (97.0–97.7)	93.9 (93.4–94.4)	<0.001
N1	90.8 (89.9–91.8)	84.6 (83.5–85.6)	<0.001	97.4 (96.9–97.9)	94.9 (94.3–95.5)	<0.001
Age						
≤50	94.7 (93.8–95.6)	92.6 (91.7–93.5)	<0.001	99.1 (98.7–99.4)	98.6 (98.1–99.0)	0.040
>50	93.0 (92.4–93.5)	86.1 (85.5–86.8)	<0.001	96.8 (96.5–97.2)	93.0 (92.5–93.5)	<0.001
Tumor subtype						
HR−/HER2+ (HER2‐enriched)	93.5 (91.8–95.3)	87.3 (85.1–89.6)	<0.001	97.9 (96.8–98.8)	95.9 (94.4–97.1)	0.009
HR+/HER2− (Luminal A)	94.3 (93.8–94.8)	89.3 (88.7–90.0)	<0.001	97.2 (96.8–97.6)	94.0 (93.5–94.5)	<0.001
HR+/HER2+ (Luminal B)	95.9 (94.9–96.9)	90.1 (88.7–91.6)	<0.001	97.8 (97.0–98.5)	95.4 (94.3–96.4)	<0.001
HR−/HER2− (Triple‐negative)	86.6 (85.0–88.3)	76.5 (74.6–78.4)	<0.001	97.4 (96.6–98.1)	93.7 (92.6–94.7)	<0.001

Abbreviation: BCT, breast‐conserving therapy.

## 4 DISCUSSION

Since the NSABP B‐06 trial, the BCT has been proven to have the same survival rate as mastectomy. Furthermore, the number of early‐stage breast cancer patients who underwent mastectomy decreased. In recent studies, however, it has been reported that breast cancer patients are having more mastectomy surgeries.^10^ In part, the increased use of mastectomy can be attributed to the perception that patients with unfavorable factors, such as younger age, HER2‐amplified breast cancer, and advanced reconstructive surgery, have a poorer prognosis.^11^ A real‐world analysis of our study showed that patients treated with BCT had better overall survival rates than those treated with a mastectomy. In addition, these results were confirmed after accounting for variables in the matched cohort. It is consistent with the results of de Boniface J et al.^12^ that the BCT yielded a better survival than mastectomy after adjusting for comorbidities and socioeconomic background.

This was a population‐based study to examine whether BCT might be more beneficial than mastectomy for different subtypes of breast cancer. We agree with previous studies that advocate BCT. Agarwal S et al.^5^ found that BCT reduced the mortality risk in patients with tumors less than 4 cm and matching nodes. And Hwang et al.^13^ demonstrated that BCT was associated with a lower risk of death. A recent study including early‐stage breast cancers also reported that the patients with BCT experienced better OS than those with the mastectomy.^14^ Several small population‐based studies conferred the same results.^15^, ^16^

However, in many populations, the studies failed to match important factors like tumor subtypes and age. Using the PSM method to compare two closely matched populations allows us to simulate randomization on the survival results of BCT and mastectomy. Regardless of tumor subtypes, tumor stage, and lymph node stage subgroups, BCT had a better overall survival and cancer‐specific survival rate than mastectomy in our study.

It is unclear why our study and those mentioned above indicate different survival outcomes, whereas several clinical trials have demonstrated equivalent survival between patients who undergo BCT and mastectomy in early‐stage breast cancer patients. There are several plausible explanations. One is that the radiation‐induced cardiotoxicity of older radiation techniques may obscure the benefit of BCT. The benefit of BCT is possibly related to incidental irradiation of lymphatics in patients with a high recurrence score and to improve three‐dimensional conformal planning.^17^, ^18^ In clinical practice, some patients with mastectomy with clinically uninvolved lymph nodes may not receive additional lymph node irradiation.^19^ The abscopal effects of radiation may be another possible reason for improved OS in patients with BCT. It has unique biological properties that inhibit the migration of tumor cells to distant organs and elicit an antitumor immune response in breast cancer patients.^20^ Furthermore, radiation can induce immunogenic cell death, which involves the release of signals and various cytokines to modify tumors’ microenvironment.^21^ It is reported that patients with *BRCA1* and *BRCA2* variant carriers treated with BCT had similar OS compared with those underwent mastectomy. For noncarriers, patients with BCT had better OS than those with mastectomy.^22^ The different expression of *BRCA1* and *BRCA2* maybe one of the explanations. It deserves further investigations in the future.

The young age of patients is well‐known as a predictor of local recurrence following BCT. Numerous studies have shown that patients under 50 years of age tend to have more aggressive lesions with a higher risk of recurrence. Recent studies of patients under 40 showed that patients in the BCT group demonstrated better OS and BCSS than those in the mastectomy group.^23^, ^24^ However, a previous study reported that the OS and distant relapse‐free survival were similar between the BCT and mastectomy group.^25^ In our research, the improved OS and BCSS result still exist in the BCT group in young patients. There is a need to perform a longer‐term study in order to determine whether BCT and mastectomy produce different effects in young women.^26^

Surgical decision‐making for breast cancer is unique in that different patient‐selected options are available with similar outcomes based on patients’ own goals and viewpoints. The changing landscape of systemic therapies and the growing understanding of patient subgroups may affect the effectiveness of local therapies. If a physician believes that a treatment will not lead to an improved outcome, he will be less likely to follow the treatment recommendations. In our study, BCT had superior survival results compared with mastectomy, even in matched patients. To reduce confounding, patients were matched 1:1 regarding variables associated with surgery type choice and survival. This means that each mastectomy patient has an exactly matched BCT case with the similar tumor characteristics. In the matched cohort, the improved OS and BCSS results for the BCT group remained significant.

Our study has some limitations. First, the SEER database did not provide local‐regional recurrence data, the irradiated technique details, and scope details. Second, we excluded medical cases with missing data on tumor characteristics and loss of follow‐up. Recurrence score (RS) is involved in treatment decisions in ER‐positive, HER2‐negative, and node‐negative breast cancer, but we have no information about RS in our study. Our study is limited by its retrospective design and the inherent potential for selection bias. In order to minimize the impact of potential bias, we analyzed the data of all eligible patients, and match‐related available factors and conducted the analysis in propensity‐matched samples. In SEER database, patients with no evidence of radiotherapy or chemotherapy found in the medical records was categorized as no/unknown. There were biases associated with unmeasured reasons for receiving or not receiving chemotherapy or radiotherapy in our analysis. Furthermore, our study is limited by the short‐term follow‐up for patients with tumor subtype because HER2 data were not available until 2010.

## 5 CONCLUSIONS

Among early‐stage breast cancer patients, we found that BCT is associated with improved overall survival and cancer‐specific survival compared with mastectomy. Although not a prospective randomized trial, it adds to growing evidence that BCT is beneficial for this population. Further investigation is needed to determine what factors contribute to efficacy. It seems advisable to encourage patients to receive BCT rather than mastectomy in early‐stage patients when feasible and appropriate.

## CONFLICT OF INTEREST

The authors declare that they have no conflict of interest.

## AUTHOR CONTRIBUTIONS

Jiali Ji, Lei Yang, and Xuexin He designed the study. Jiali Ji, Shushu Yuan, Jiawei He, and Hong Liu collected and analyzed the data. Jiali Ji, Shushu Yuan, and Jiawei He contributed to the manuscript drafting. Hong Liu, Lei Yang, and Xuexin He critically revised the manuscript. All authors approved the manuscript version to be published.

## ETHICS APPROVAL AND CONSENT TO PARTICIPATE

This article does not contain any studies with human participants or animals performed by any of the authors.

## Supporting information

Fig S1‐S4

## Data Availability

Our study data were publicly available in the Surveillance, Epidemiology and End Results, https://seer.cancer.gov.
