# Supplementary material for: Breast‐conserving therapy is associated with better survival than mastectomy in Early‐stage breast cancer: A propensity score analysis
Source: Cancer Med. 2022 Feb 25;11(7):1646–58. doi: 10.1002/cam4.4510 (PMC8986144; doi:10.1002/cam4.4510)

**Supplementary Figures**

**Figure legends**

**Figure S1: Overall survival (OS) rate of patients underwent BCT and mastectomy in all eligible patients stratified in tumor subtype.**

A: OS rate of patients underwent BCT and mastectomy in HER2+ breast cancer

B: OS rate of patients underwent BCT and mastectomy in Luminal A breast cancer

C OS rate of patients underwent BCT and mastectomy in Luminal B breast cancer

D: OS rate of patients underwent BCT and mastectomy in Triple-negative breast cancer

**Figure S2: Overall survival (OS) rate of patients underwent BCT and mastectomy in all eligible patients stratified in the different age groups.**

A: OS rate of patients underwent BCT and mastectomy aged ≤50 years old breast cancer

B: OS rate of patients underwent BCT and mastectomy aged >50 years old breast cancer

**Figure S3: Overall survival (OS) rate of patients underwent BCT and mastectomy in all patients stratified in different tumor and node stage.**

A: OS rate of patients underwent BCT and mastectomy in T1 stage breast cancer

B: OS rate of patients underwent BCT and mastectomy in T2 stage breast cancer

C OS rate of patients underwent BCT and mastectomy in N0 stage breast cancer

D: OS rate of patients underwent BCT and mastectomy in N1 stage breast cancer

**Figure S4: Overall survival (OS) rate of patients underwent BCT and mastectomy in propensity-score matching (PSM) cohort stratified in different tumor and node stage.**

A: OS rate of patients underwent BCT and mastectomy in T1 stage breast cancer

B: OS rate of patients underwent BCT and mastectomy in T2 stage breast cancer

C OS rate of patients underwent BCT and mastectomy in N0 stage breast cancer

D: OS rate of patients underwent BCT and mastectomy in N1 stage breast cancer

**Figure S1:**


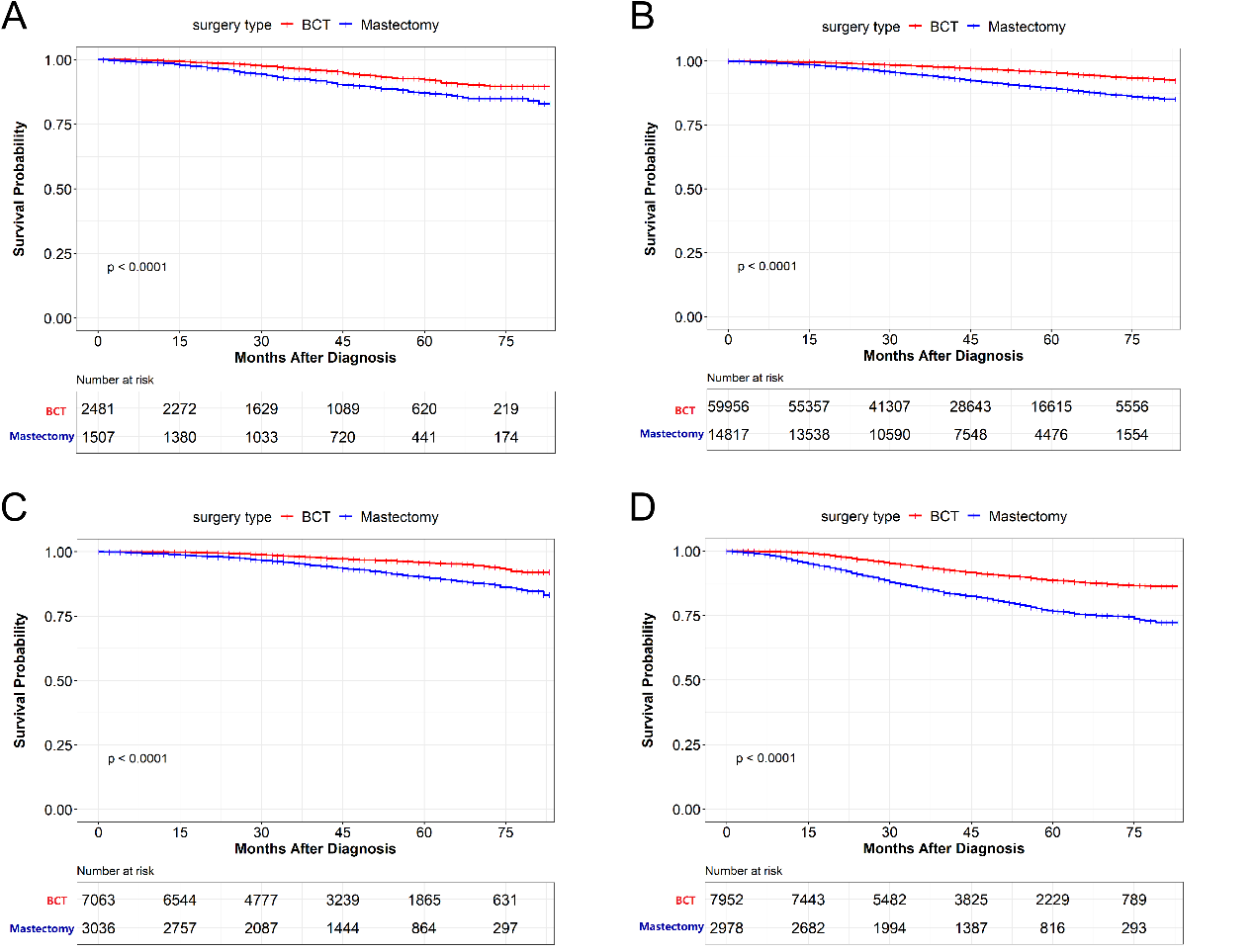


**Figure S2:**


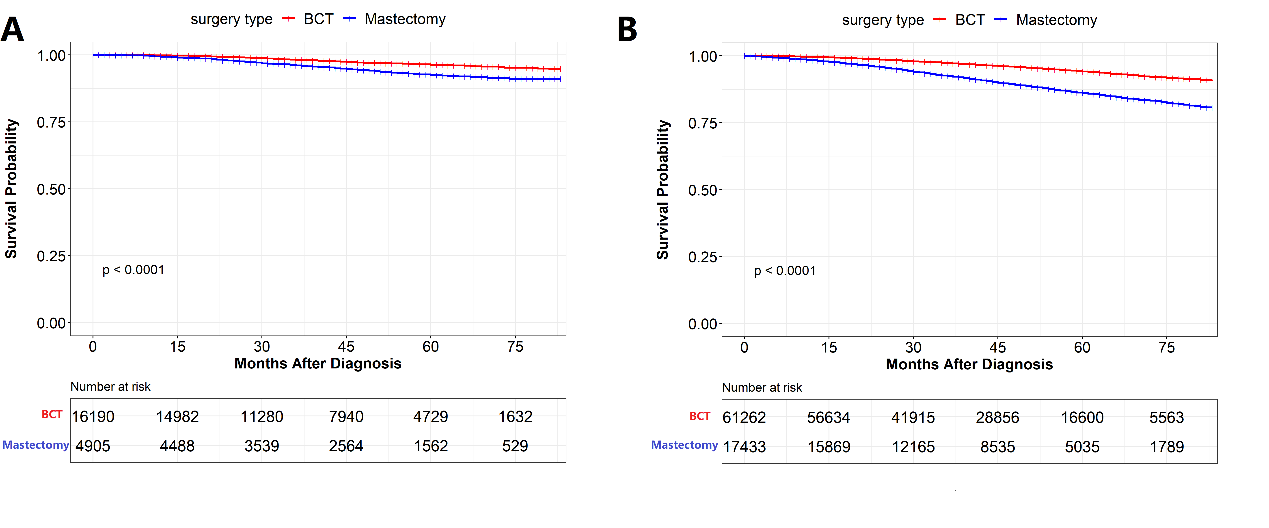


**Figure S3:**


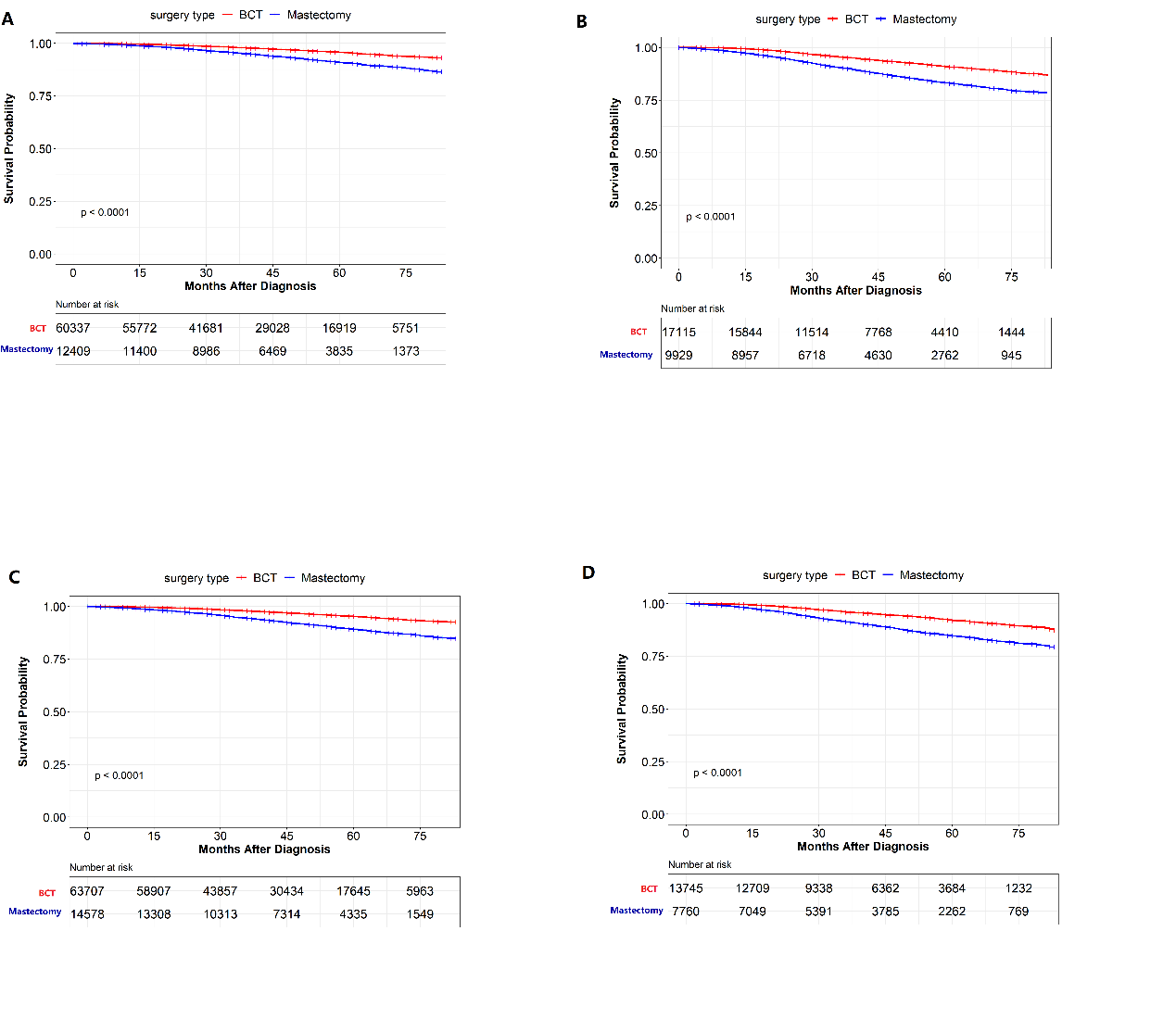


**Figure S4:**


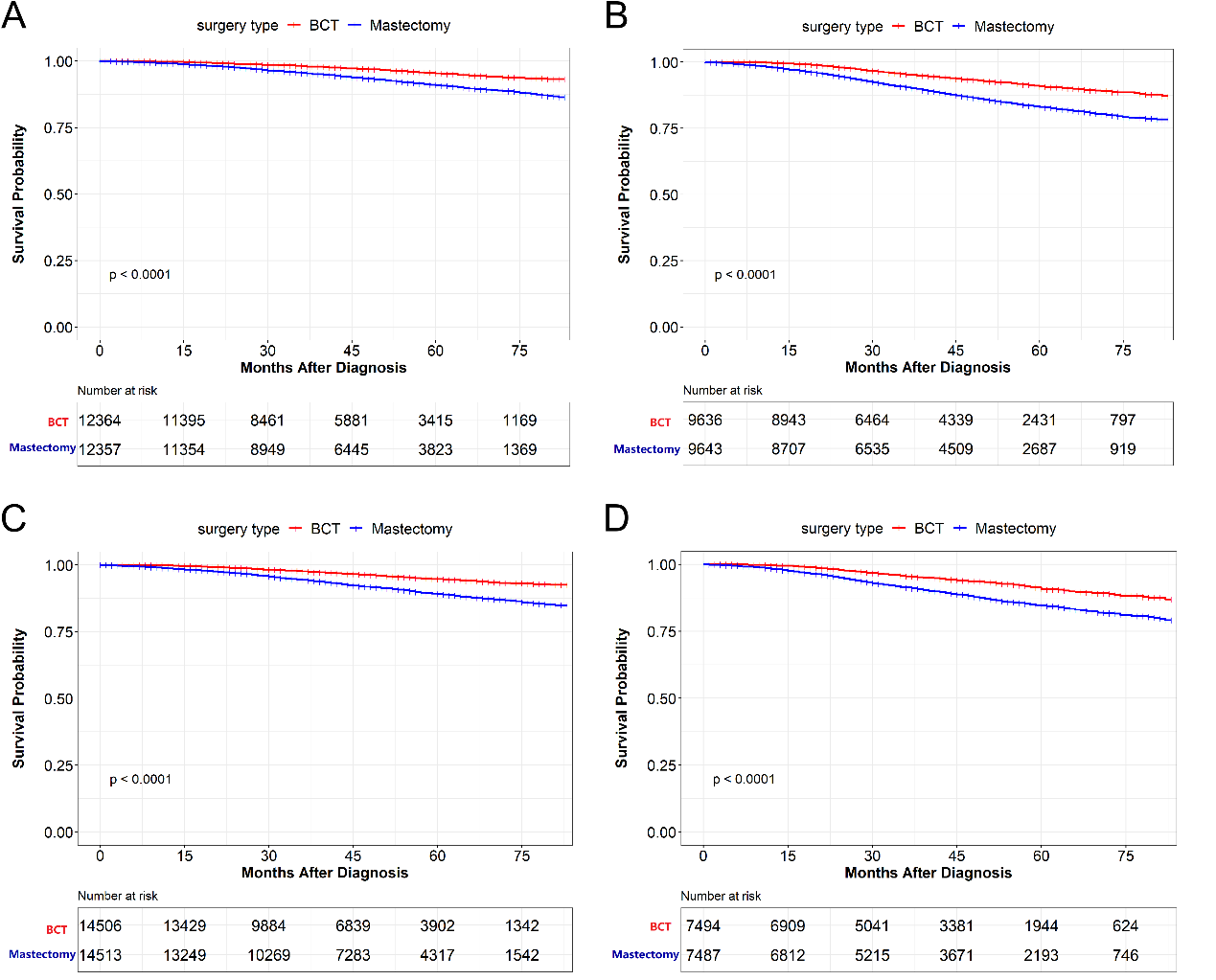

Supplement: Supplementary file 1 — Fig S1‐S4 [file CAM4-11-1646-s001.docx]
